# How to make a protective face shield or visor

**Published:** 2020-09-01

**Authors:** Dupe Ademola-Popoola, Fiona Lloyd

**Affiliations:** 1 Reader & Consultant Ophthalmologist: University of Ilorin & University of Ilorin Teaching Hospital, Nigeria.; 2 Former Intensive Treatment Unit Nurse, Hertfordshire, UK.

**Face shields, or visors, protect the eyes, nose and mouth against droplet transmission of respiratory viruses such as COVID-19. If they are unavailable, or too expensive, here is how to make your own.**

There is an increasing recognition of the importance of eye protection in reducing the risk of COVID-19 transmission and infection among health care workers, and health care workers are urged to wear eye protection when in close contact with patients.^1^,^2^ Face shields, or visors, offer more protection than a mask and safety goggles, as they prevent viral droplets from landing on the face, from where they can more easily be transferred to the eyes, nose or mouth. However, face shields are not always available locally.

It is possible to make an eye shield using locally available materials. Face shields do not need to be sterilised, but they must be cleaned between patients. Clean with detergent first, then using hospital disinfectant. Finish by wiping with water or 70% ethanol to remove any residues.

**Top tip:** To avoid the shield (or your spectacles) misting up when you wear an ordinary surgical mask, tape the upper edge of the mask to your cheeks and the bridge of your nose using surgical or micropore tape.

## Face shield with foam rest

For health workers who need to wear a face shield for a long period of time, we recommend this type, which has a foam rest to improve comfort on the forehead.

**You will need:**

- Transparent, flexible plastic film (or a clear binder)
- Strips of cloth, approximately 3–4 cm wide (Figure 1), a woven belt (Figure 2) or a broad elastic band
  **Figure 1:**
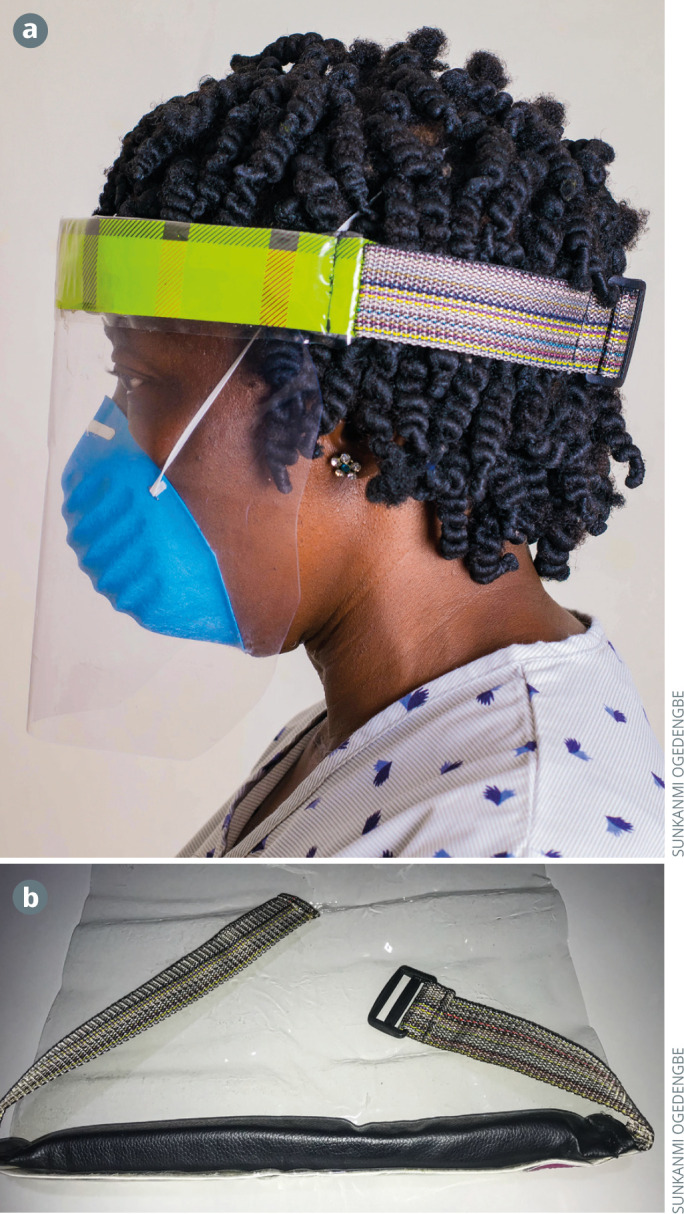
**a.** Face shield made from a fabric belt. **b**. The soft foam or cotton wool is wrapped in water-resistant material
  **Figure 2:**
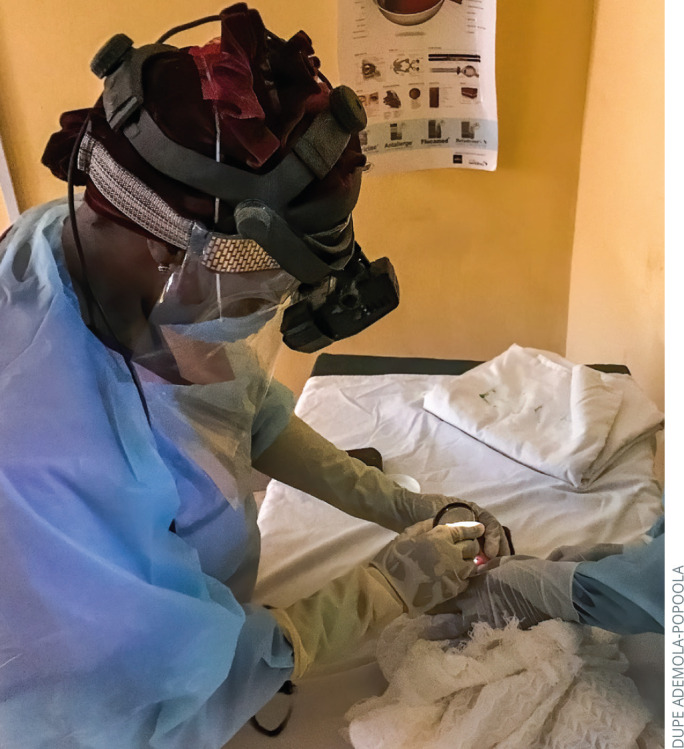
Face shield used during indirect ophthalmoscopy examination
- Soft foam or cotton wool
- A stapler, or needle and thread/twine or adhesive glue/gum
- Scissors

### Method

1. Take these measurements to make a customised face shield:
  - From temple to temple, across the forehead (about 28–35 cm)
  - While looking straight ahead, the distance from brow area to below the chin (about 21–25 cm, depending on the length desired)
  - The head circumference at the widest point across the forehead (about 55–63 cm)
2. Cut the transparent, flexible film into a rectangle:
  - Width equal to **a** (the distance from temple to temple, across the forehead)
  - Height equal to **b** (the distance from the brow to below the chin, or as desired)
3. Cut the strips of cloth, woven belt or elastic band as follows:
  - Strips of cloth: two lengths of 30–40 cm (long enough to tie behind the head)
  - Woven belt: **c** (the head circumference) + approximately 15 cm if a belt buckle is used, or longer if it will be tied
  - Elastic band: **c** (the head circumference) + approximately 5 cm (long enough to stitch or staple together behind the head).
4. The transparent film will fit sideways across the face. Hold it horizontally, so the shorter edges are on either side, and place on a flat, clean surface in front of you. Attach the strips of cloth, belt or elastic to the upper edge (or upper corners) using adhesive glue or gum, or by stitching or stapling them, as follows:
  - Strips of cloth: attach a strip to each of the two upper corners of the face shield and tie them together behind the head.
  - Woven belt: attach across the top of the face shield, leaving extra length on both sides so it can be secured using a belt buckle or by tying a knot at the back of the head. If the belt is too short, cut it in half and attach it to the two upper corners.
  - Elastic band: attach across the upper edge of the face shield, then bring the ends around behind the shield and sew or staple them together to form a loop. The amount of overlap depends on how tight a fit the person wants. **Note:** If you are using an elastic band, be aware that it may not fit if you wear your hair in a different style. If you change your hairstyle regularly, it would be better to use a woven belt or strips of cloth that tie at the back.
5. Cut soft foam or cotton wool in a strip, long enough to fit across the forehead (measurement **a**) and about 4 cm wide. Glue, staple or stitch this to the inside of the transparent film. If possible, cover the foam or cotton wool with a water-resistant material; this allows it to be wiped clean easily, especially if the wearer is sweating while working in a warm climate or in a warm health care environment.

The soft foam serves as a spacer and provides comfort for the forehead, makes breathing easier, and reduces mist formation if it has to be worn over a long period of time, e.g., for consultations, indirect ophthalmoscopy, or laser procedures.

## Face shields for paediatric ophthalmologists

Children are not able to practice social distancing or respiratory hygiene, which is cause for concern amongst paediatric ophthalmologists. For this group of eye health workers, a face shield without a foam rest is comfortable enough, as they may only wear the shield for short periods of time.

To make this type of face shield, you will need:

- Transparent, flexible plastic film, A4 size (21 cm x 30 cm)
- A hole punch
- Elastic band (30–40 cm in length)

**Note:** It is possible to create clear plastic film by putting an empty A4 laminating pouch through an office laminating machine.

### Method

- The A4 plastic film fits sideways across the face, so the width (30 cm) covers the area from the forehead to below the chin (Figure 3).
  **Figure 3:**
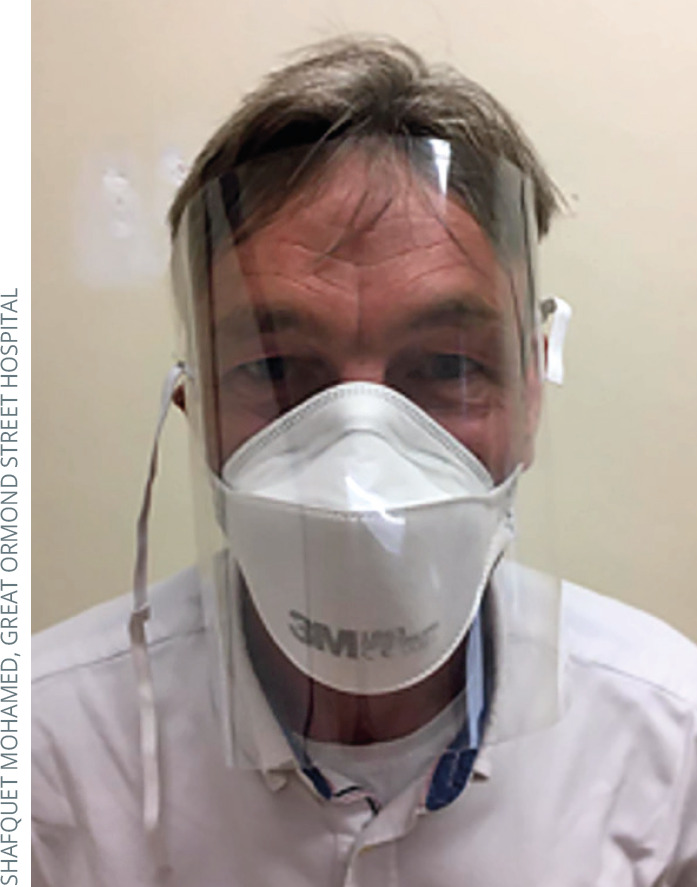
An A4 plastic sheet covers the face from the forehead to below the chin
- Use the hole punch to create two holes on either side of the A4 plastic film when held horizontally, approximately 5 cm from the top and 1 cm from the side.
- Thread elastic band through both holes and tie at one end.
- Leave the other side untied until the individual tries it on and adjusts the elastic band to fit them (Figure 4).
  **Figure 4:**
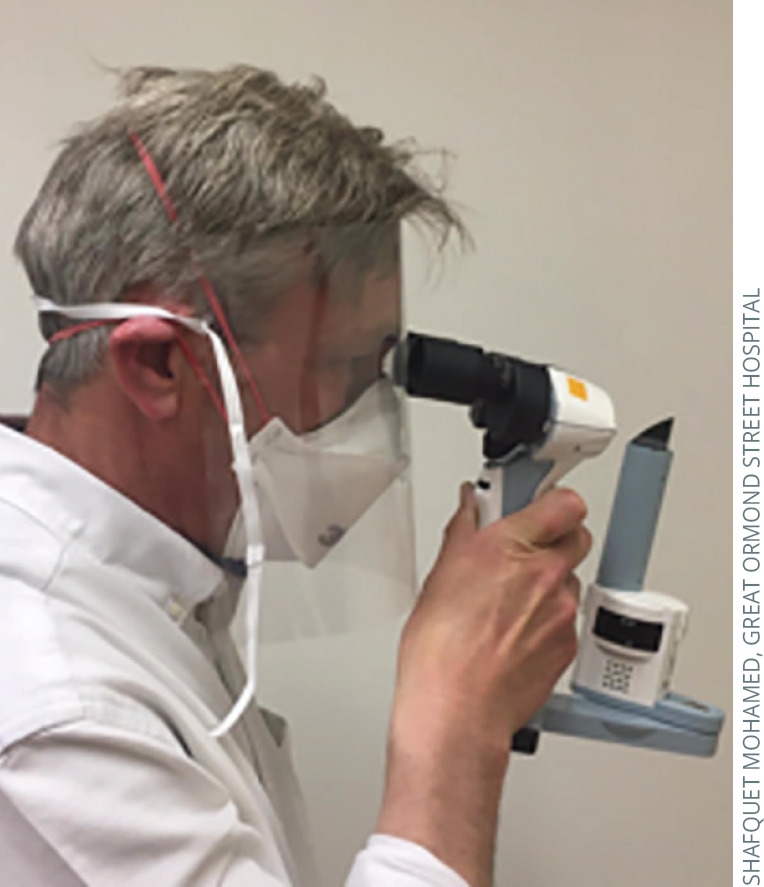
A hole punch is used to create small openings on either side, and a length of elastic is used to keep it in place

**Figure 5:**
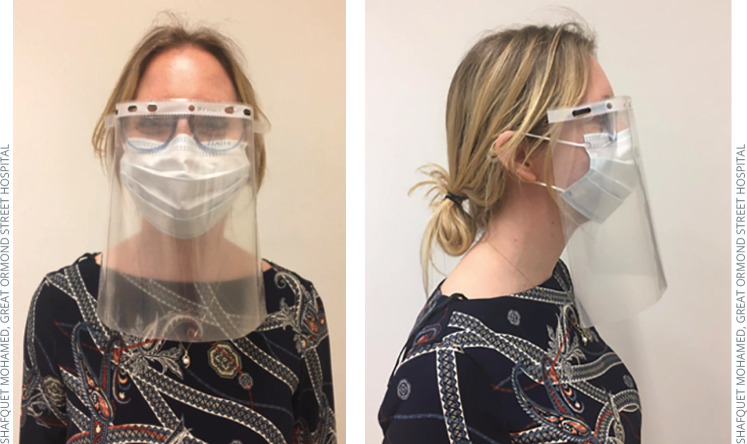
A simple face shield for spectacle wearers

For spectacle wearers, a quick and easy solution is to use a clear plastic binder pocket, or sleeve, and thread the arms of your spectacles through the holes, as shown in Figure 5.
